# Additive Antiproliferative and Antiangiogenic Effects of Metformin and Pemetrexed in a Non-Small-Cell Lung Cancer Xenograft Model

**DOI:** 10.3389/fcell.2021.688062

**Published:** 2021-06-21

**Authors:** Jiun-Long Wang, Ying-Wei Lan, Yi-Ting Tsai, Ying-Cheng Chen, Theresa Staniczek, Yung-An Tsou, Chih-Ching Yen, Chuan-Mu Chen

**Affiliations:** ^1^Ph.D. Program in Translational Medicine, Department of Life Sciences, National Chung Hsing University, Taichung, Taiwan; ^2^Division of Chest Medicine, Department of Internal Medicine, Taichung Veterans General Hospital, Taichung, Taiwan; ^3^Division of Endocrinology and Metabolism, Department of Internal Medicine, Taichung Veterans General Hospital, Taichung, Taiwan; ^4^Department of Dermatology, Venereology and Allergology, Center of Excellence in Dermatology, University Medical Center and Medical Faculty Mannheim, Heidelberg University, Mannheim, Germany; ^5^Department of Otolaryngology-Head and Neck Surgery, China Medical University Hospital, Taichung, Taiwan; ^6^Department of Internal Medicine, China Medical University Hospital, College of Health Care, China Medical University, Taichung, Taiwan; ^7^The iEGG and Animal Biotechnology Center, Rong Hsing Research Center for Translational Medicine, National Chung Hsing University, Taichung, Taiwan

**Keywords:** metformin, pemetrexed, lung cancer, angiogenesis, orthotopic xenograft, chorioallantoic membrane

## Abstract

Lung cancer is heterogeneous and challenging to cope with once it has progressed. Chemotherapy is the first step once no active driver mutation has been discovered. Non-antitumor drugs have been found to be beneficial when used as adjuvants to chemotherapy. In this study, the additive effect and mechanism of metformin combined with pemetrexed in non-small-cell lung cancer (NSCLC) cells were elucidated. Three NSCLC cell lines, A549, H1975, and HCC827, were used to analyze tumor cell proliferation, colony formation and the cell cycle *in vitro* when exposed to metformin alone, pemetrexed alone or their combination. We found that combination treatment in three cell lines exerted antiproliferative effects through cell cycle arrest in the S phase. An *ex vivo* chicken chorioallantoic membrane (CAM) assay was used to examine the antiangiogenic effect of metformin combined with pemetrexed on vascular structure formation. We further created an A549 orthotopic xenograft model with an *in vivo* imaging system (IVIS) and explored the associated indicators involved in the tumorigenic process. The *in vitro* results showed that the combination of metformin and pemetrexed exhibited an antiproliferative effect in reducing cell viability and colony formation, the downregulation of cyclin D1 and A2 and the upregulation of CDKN1B, which are involved in the G1/S phase. For antiangiogenic effects, the combination therapy inhibited the vascular structure, as proven by the CAM assay. We elucidated that combination therapy could target VEGFA and Endoglin by RT-qPCR, ELISA and histopathological findings in an A549 orthotopic NSCLC xenograft model. Our research demonstrated the additive antiproliferative and antiangiogenic effects of the combination of metformin with pemetrexed in NSCLC and could be applied to clinical lung cancer therapy.

## Introduction

Lung cancer remains a challenging obstacle and ranks as the leading cause of mortality worldwide, including in Taiwan (Hsu et al., 2015; Siegel et al., 2017; Tseng et al., 2019). The most common type of lung cancer is non-small-cell lung cancer (NSCLC), which accounts for up to 85% of all cases. Lung adenocarcinoma is predominant among NSCLC, and most patients are diagnosed with an advanced or metastatic status (Levy and Doyen, 2018). Once driver mutations, such as those in epidermal growth factor receptor (EGFR) or echinoderm microtubule-associated protein-like 4-anaplastic lymphoma kinase (EML4-ALK) fusion mutations, are detected, targeted therapy can be applied (Hsu et al., 2015; Mok et al., 2017; Soria et al., 2017). Discovery of the expression of programmed death-1 (PD-1) or PD-ligand 1 (PD-L1) makes the patient a candidate for the use of immune checkpoint inhibitors (ICIs) (Reck et al., 2016, 2019; Berghmans et al., 2020; Satouchi et al., 2020). When patients do not harbor active driver mutations or immune checkpoint expression, chemotherapy is the mainstream treatment.

Among chemotherapeutic agents, pemetrexed (Alimta^®^) is chosen for front-line therapy in advanced or metastatic NSCLC (especially for lung adenocarcinoma) in a single use for maintenance or combined with platinum for first line therapy (Scagliotti et al., 2008; Belani et al., 2012a, b). Pemetrexed is an antineoplastic agent and has an antifolate effect. A 3-week therapy is arranged in clinical practice. Maintaining chemotherapy efficacy and overcoming drug resistance are essential. Because a great amount of time and money is required to develop new drugs (Saxena et al., 2015), repositioning or repurposing present non-antitumor drugs for potential antitumor effects has drawn much attention, and an increasing number of studies have been devoted to this idea.

Metformin was first used as an oral antidiabetic agent (OAD) and is widely used for sugar control. It inhibits gluconeogenesis and reduces insulin resistance, which is essential for tumor growth (Pawalowska and Markowska, 2012). Based on earlier observation studies, diabetic patients under metformin treatment were observed to have a lower cancer incidence, with an up to 23% reduction rate compared with other OAD groups (Evans et al., 2005; Currie et al., 2012) and an approximately 39∼45% reduction in the Taiwanese population (Lai et al., 2012). Recent observational studies showed that metformin use is beneficial for the lung cancer population (Lin et al., 2015; Arrieta et al., 2016; Chuang et al., 2018; Brancher et al., 2020; Kang et al., 2021). Dosage and time dependent effects have been extensively shown. In preclinical studies, metformin demonstrates additive effects when combined with antitumor reagents and can overcome drug resistance and reduce chemotherapeutic dosages (Kato et al., 2012; Lin et al., 2013; Tseng et al., 2013; Fatehi Hassanabad and MacQueen, 2020).

Metformin can activate adenosine monophosphate-activated protein kinase (AMPK) and negatively regulate the mammalian target of rapamycin (mTOR) pathway. The AMPK pathway is supported by liver kinase B1 (LKB1) (Zakikhani et al., 2006; Sinnett-Smith et al., 2013). The mTOR pathway is responsible for cell viability, protein synthesis and proliferation. Targeting the mTOR pathway halts cell proliferation and growth. When metformin is used alone or combined with chemotherapeutic agents, more theories have been proposed (Peng et al., 2017; Levy and Doyen, 2018; Fatehi Hassanabad and MacQueen, 2020). Luo et al. (2019) found that metformin induces apoptosis by promoting survivin degradation through the AMPK/PKA (protein kinase A)/GSK (glycogen synthase kinase)-3B pathway in NSCLC. Other research showed that the combination of metformin with cisplatin leads to decreased expression of vascular endothelial growth factor-C (VEGF-C) and VEGF receptor-3 (VEGFR-3) at the protein and mRNA levels (Chen and Chen, 2015).

Few studies have focused on the antitumor mechanism induced by metformin combined with pemetrexed. Although a lung cancer cell line *in vitro* study performed by Zhang et al. (2017) proposed antiproliferative and apoptosis processes, there is still a lack of *in vivo* studies and real-time imaging monitoring to expand and elucidate the possible mechanism besides the AMPK-dependent pathway. Therefore, we set up serial experiments and platforms in *in vitro*, *ex vivo* and *in vivo* models to identify potential additive and novel therapeutic targets, such as angiogenesis, in combination therapy with metformin and pemetrexed in non-small-cell lung cancer.

In this study, the combination therapy of metformin with pemetrexed was designed to test the additive effect of antiproliferation on three NSCLC cell lines through the study of cell viability, colony formation and cell cycle functions. The additive antiangiogenic effect was proven by a chicken chorioallantoic membrane (CAM) assay and an orthotopic NSCLC xenograft model. The expression of VEGF-associated angiogenic indicators (VEGF and Endoglin) and the tumor marker carcinoembryonic antigen (CEA) was also determined for a mechanistic study.

## Materials and Methods

### Chemicals

Metformin hydrochloride and crystal violet were purchased from Sigma-Aldrich (St. Louis, MO, United States). Pemetrexed was obtained from Eli Lilly and Company (Indianapolis, IN, United States).

### Cell Lines

Human lung adenocarcinoma cell lines A549 (ATCC CCL-185), H1975 (ATCC CRL-5098), and HCC827 (ATCC CRL-2868) were purchased from the American Type Culture Collection (Manassas, VA, United States), and an A549 stable dual fluorescent protein-expressing cell line (A549-iRFP-2A-Venus) was established previously (Lai et al., 2016). Normal human embryonic lung cell line WI-38 VA-13 subline 2RA (WI38-2RA; BCRC-60504) was purchased from Bioresource Collection and Research Center (Hsinchu, Taiwan). Both A549 cell lines were maintained in Ham’s F12K medium (Life Technologies; Waltham, MA, United States); H1975 and HCC827 cell lines were maintained in RPMI1640 medium (Life Technologies); WI38-2RA cell line was maintained in Eagle’s minimal essential medium (Life Technologies). All culture medium was supplemented with 10% fetal bovine serum (FBS; Life Technologies), 2 mM L-glutamine (Life Technologies) and 1% penicillin/streptomycin (Life Technologies), and were incubated at 37°C in a 5% CO_2_ incubator.

### Cell Viability MTT Assay

A549, H1975, and HCC827 cells were seeded in 24-well culture plates (5 × 10^4^ cells; BD Biosciences, La Jolla, CA, United States) and incubated overnight to allow adhesion. The cells were treated with metformin (Met; 0.2 mM), pemetrexed (Pem; 1 μM), or both (0.2 mM Met + 1 μM Pem) for 48 h. The viable cells were stained and detected using the MTT [3-(4,5-dimethylthiazol-2-yl)-2,5-diphenyltetrazolium bromide] staining method at a wavelength of 540 nm by a Multiskan GO spectrophotometer (Thermo Fisher Scientific; Waltham, MA, United States) as previously described (Lan et al., 2015).

### Cell Proliferation and Colony-Forming Unit Assay

A549, H1975, and HCC827 cells were seeded at 1,000 cells/well in 12-well culture plates (BD Biosciences) and incubated overnight to allow adhesion. The cells were treated with metformin (Met; 0.2 mM), pemetrexed (Pem; 1 μM), or both (Met; 0.2 mM + Pem; 1 μM), and the cultures had a replacement of fresh drug and culture medium every 2 days. After 10 days in culture, the cells were washed with DPBS, fixed with 10% formalin for 2 min, and stained with 0.5% crystal violet (dissolved in methanol) for 20 min at room temperature. The crystal violet solution was removed, after which the cells were washed with DPBS at least twice to get a clear background. Representative photographs were captured using a camera, and the quantification of the crystal violet-positive staining was performed using NIH Image software (ImageJ 1.35 d; NIH, Bethesda, MD, United States).

### Cell Cycle Analysis

A549, H1975, and HCC827 cells were treated with metformin (0.2 mM) and/or pemetrexed (1 μM) for 48 h. The cells were harvested and stained as previously described (Hung et al., 2014). The cells were fixed with 70% ice-cold ethanol and incubated at −20°C for 1 h. The fixed cells were centrifuged and the pellets were resuspended in lysis buffer (0.5% Triton X-100 and RNAse A at 10 μg/ml) (Life Technologies) at 37°C for 40 min. The cells were stained with 50 μg/ml propidium iodine (Sigma-Aldrich, St. Louis, MO, United States). After 20 min of incubation at 4°C in the dark, the stained cells were analyzed using an Accuri C6 Plus flow cytometer (BD Biosciences). The percentages of cells in the G1, S, and G2/M phases were calculated using Accuri C6 Plus software (BD Biosciences).

### FITC Annexin V Apoptosis Detection Assay

A549, H1975, and HCC827 cells were treated with metformin (0.2 mM) and/or pemetrexed (1 μM) for 48 h. The cells were harvested and stained according to the manufacturers’ protocols (BD Biosciences). The cells were analyzed using Accuri C6 Plus flow cytometer (BD Biosciences). The percentage of apoptotic cell in each quadrant was calculated using Accuri C6 Plus software (BD Biosciences).

### Western Blot Analysis

Western blot analysis was performed as previously described (Lan et al., 2019). The antibodies used were anti-phosphoAMPK (Thr172), anti-AMPK, anti−phosphoAkt (Ser473), anti-panAkt, anti-CDKN1B (Cell Signaling Technology, Danvers, MA, United States), anti-cyclin D1 (Abcam, Cambridge, MA, United States), and anti-β-actin (Novus Biologicals, Littleton, CO, United States) antibodies.

### Chicken Chorioallantoic Membrane Assay

The effect of metformin and pemetrexed on angiogenesis was evaluated using the CAM assay, following a method with minor modification. Fertilized chicken eggs were maintained in a humidified egg incubator at 37°C. After 3 days of incubation, a small hole was made using an 18-gauge needle to extract 4 ml of albumin from the egg. An approximate 1-cm^2^ window was created to check the health and normal growth of the embryos and to exclude malformations or dead embryos. After fenestration, the windows were sealed with tape to prevent contamination and placed back into the incubator. On day 7 of the incubation, 5 × 10^6^ A549 cells suspended in 30 μl of 50% Matrigel (BD Biosciences) were directly seeded on the membrane of CAM through the small window created earlier. The cells were then treated with 40 μl metformin (Met; 0.1 mg/egg), pemetrexed (Pem; 0.01 mg/egg), or both (Met; 0.1 mg/egg + Pem; 0.01 mg/egg) for 48 h, and the cultures had a replacement of fresh drug and culture medium every 2 days. After cell grafting and treatment, the windows were covered again, and the eggs were placed back into the incubator. After a 7-day period of treatment, each egg was observed, and the blood vessels were photographed. The antiangiogenic effects of metformin and pemetrexed on the CAMs were quantified, and the mean vessel area was quantified as a percentage of the total area (vessel density), total vessel length and the number of branch points, which were estimated using WimCAM software (Onimagin Technologies, Cordoba, Spain).

### RNA Isolation and RT-qPCR

Total RNA was isolated from at least two FFPE lung tissue blocks, as previously described (Körbler et al., 2003). Briefly, sections were deparaffinized by immersion in xylene twice for 15 min at room temperature with agitation, followed by two rounds of centrifugation for 2 min at 10,000 × *g*. Then, rehydration steps were performed (rinsing twice in 100% ethanol and once in 95, 80, and 60% ethanol). After each step, the tissue was harvested by centrifugation at 10,000 × *g* for 10 min. Then, the pellets were resuspended in 500 μl digestion buffer [10 mM NaCl, 500 mM Tris (pH 7.6), 20 mM EDTA, and 1% SDS] and 500 μg/ml proteinase K and incubated at 45°C overnight. Prior to RNA purification, in some samples, we inactivated proteinase K at 100°C for 7 min. The subsequent protocol for total RNA extraction was the same as for the TRIzol reagent (Life Technologies), according to the manufacturer’s instructions (Tung et al., 2015). The mRNAs were reverse transcribed into cDNA, and RT-qPCR was performed as previously described (Lan et al., 2017). The sequences of the specific primers used are listed in Table 1. Relative gene expression was determined by the ΔΔCt method, where Ct was the threshold cycle. The relative mRNA expression levels were normalized to the housekeeping genes.

**TABLE 1:** Oligonucleotide primers used for RT-qPCR analysis.

Primer set	Oligonucleotide sequence	Size (bp)
GFP	Forward: ACC GGG GTG GTG CCC ATC CT	314
	Reverse: TTC ACC TCG GCG CGG GTC TT	
mVEGFA	Forward: CTG TGC AGG CTG CTG TAA C	184
	Reverse: ACA GTG ATT TTC TGG CTT TGT TC	
mEndoglin	Forward: CCC TCT GCC CAT TAC CCT G	120
	Reverse: GTA AAC GTC ACC TCA CCC CTT	
mGAPDH	Forward: TGA CCT CAA CTA CAT GGT CTA CA	85
	Reverse: CTT CCC ATT CTC GGC CTT G	
hCEACAM5	Forward: CTG TCC AAT GAC AAC AGG ACC	174
	Reverse: ACG GTA ATA GGT GTA TGA GGG G	
hcyclinA2	Forward: GGA TGG TAG TTT TGA GTC ACC AC	202
	Reverse: CAC GAG GAT AGC TCT CAT ACT GT	
hcyclinD1	Forward: TCT CCA AAA TGC CAG AGG CG	178
	Reverse: AGG AAG TTG TTG GGG CTC CT	
hCDKN1B	Forward: ATAAGGAAGCGACCTGCAACCG	119
	Reverse: TTC TTG GGC GTC TGC TCC ACA G	
hβ-actin	Forward: GCG AGA AGA TGA CCC AGA TC	103
	Reverse: CCA GTG GTA CGG CCA GAG G	

### Orthotopic Xenografts

Eight-week-old male nude mice (BALB/cAnN. Cg-Foxnlnu/CrlNARL) were purchased from the National Laboratory Animal Center (Taipei, Taiwan) and fed with the purified rodent diet AIN76A (Research Diets, New Brunswick, NJ, United States) to reduce autofluorescence. Orthotopic xenografts of the A549 cell suspension (1 × 10^6^ A549 cells suspended in 50% Matrigel; v/v = 1:1) were generated through transpleural injection as previously described (Lai et al., 2016). The animal experiments were approved by the Institutional Animal Care and Use Committee of National Chung Hsing University (IACUC no. 105–070) and were carried out in compliance with institutional guidelines.

### *In vivo* Imaging and Quantification of Lung Tumors

After 4 weeks of A549 cell grafting, mice were randomly selected for oral gavage with metformin (Met; 250 mg/kg/day), intraperitoneal injection of pemetrexed (Pem; 150 mg/kg/twice a week), or both (Met + Pem). The dosage and delivered route were based on previous studies (Steiner et al., 2007; Gong et al., 2020). On week 3, after drug treatment, the iRFP fluorescence was detected by an IVIS^®^ Spectrum CT instrument (PerkinElmer, Richmond, CA, United States) with Ex/Em wavelength filters set at 675/720 nm. A fixed region of interest (ROI) was selected in the mouse chest to obtain the total radiant efficiency [(p/s)/(μW/cm^2^)] values of the iRFP fluorescence and then quantified by Living Image software (PerkinElmer). Then, blood and lung tissues were collected for further examination.

### Enzyme-Linked Immunosorbent Assay

We performed Enzyme-linked immunosorbent assays (ELISAs) for the assessment of Endoglin (R&D Systems, Minneapolis, MN, United States), VEGF (Sigma-Aldrich), and CEA (Abcam) according to the manufacturers’ protocols.

### Histopathological Staining

The lung samples were fixed in 10% formalin and embedded in paraffin sections before staining with hematoxylin and eosin (Sigma-Aldrich) according to standard protocols.

### Immunofluorescent Staining

Lung samples were cut into 4 μm thick sections and taken from along the maximum area axis of the lobe. IF staining of the paraffin-fixed sections to evaluate the amount of engrafted A549-iRFP-2A-Venus cell growth *in vivo* was performed. The deparaffinized sections were blocked with 4% bovine serum albumin (Sigma-Aldrich) in PBS with 0.2% Triton X-100 for 2 h at room temperature. Sections were then incubated overnight at 4°C with an anti-VEGF-A antibody (1:40; Novus Biologicals, Littleton, CO, United States) or anti-PECAM1 antibody (1:50; Arigo Biolaboratories, Hsinchu City, Taiwan). Sections were incubated with secondary antibody (Alexa Fluor^®^ 546-conjugated goat anti-rabbit IgG) for 1 h at room temperature. After washing with PBS, the slides were stained with DAPI for nuclear counterstaining and mounted with low viscosity aqueous mount (ScyTek Laboratories, Logan, UT, United States), after which fluorescence microscopy was used for capture and analysis.

### Statistical Analysis

All data are represented as the mean ± SD. For multiple comparisons, one-way analysis of variance analysis will be used, followed by the Tukey’s *post hoc* test for analyzing parametric data. All statistical analyses will be performed using GraphPad 8.0 Prism (GraphPad Software, Inc., San Diego, CA, United States). *P* < 0.05 represents statistical significance.

## Results

### Additive Antiproliferative Effect of the Combination of Metformin and Pemetrexed in NSCLC Cell Lines

**FIGURE 1:**
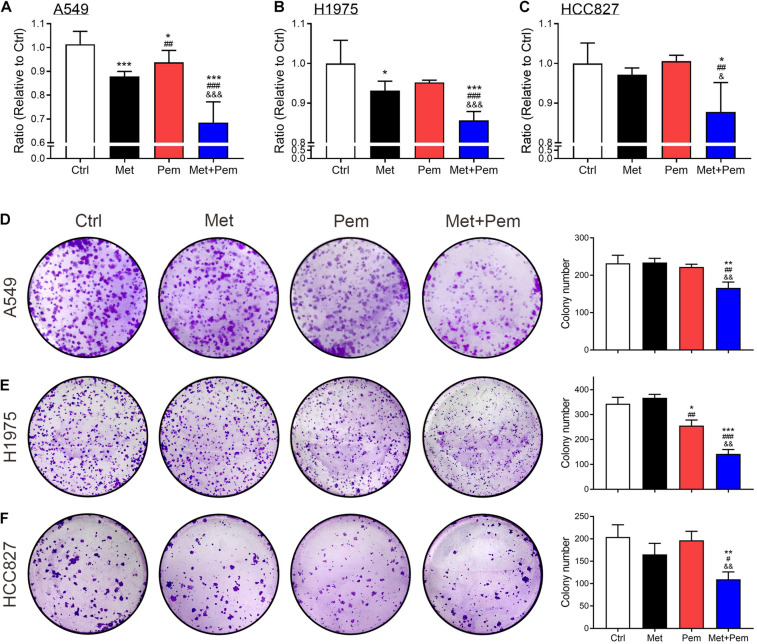
Additive antiproliferative effects of metformin and pemetrexed in the NSCLC cell lines. An MTT assay was used to detect the growth of **(A)** A549, **(B)** H1975, and **(C)** HCC827 cells treated with metformin (0.2 mM) and/or pemetrexed (1 μM) for 48 h. The ratio of the cell proliferation rate relative to control was calculated. **(D–F)** A colony formation assay was performed. Three cell lines were seeded at low density (1,000 cells/well) in 12-well plates. After 24 h of adhesion, the cells were treated with metformin and/or pemetrexed for 10 days. The colonies were stained with crystal violet. Representative photographs of each well were taken with a camera. [**(D–F)**, right panel] Quantification of the cell colonies by imaging software. The data are presented as the mean ± SD of those from three independent experiments with three technical replicates in each. *P < 0.05, **P < 0.01, and ***P < 0.001 compared with the Ctrl group; ^#^*P* < 0.05, ^##^*P* < 0.01, and ^###^*P* < 0.001 compared with the Met group; ^&^*P* < 0.05, ^&&^*P* < 0.01, and ^&&&^*P* < 0.001 compared with the Pem group.

We analyzed and confirmed the antiproliferative function of metformin alone (0.2 mM), pemetrexed alone (1 μM) or a combination in the A549, H1975, and HCC827 cell line model by the MTT method. After 48 h of treatment, the use of metformin or pemetrexed alone showed significant decreases in A549 and H1975 cell number compared to the control group, but there was a more significant reduction in the metformin and pemetrexed combination group (Figures 1A,B). The HCC827 cell number showed no change between control and the use of metformin or pemetrexed alone groups, but there was a significant reduction in the metformin and pemetrexed combination group (Figure 1C). We also checked the colony formation units (CFUs) in the four groups in three cell lines and quantitatively analyzed them (Figures 1D–F). Three cell lines showed similar results that much less colony formation, an approximately 50% reduction, in the metformin and pemetrexed combination group than in the control and single drug groups.

### Additive Cell Cycle Arrest Effect of the Combination of Metformin and Pemetrexed in NSCLC Cell Lines Through Modulating AMPK-AKT Signaling

**FIGURE 2:**
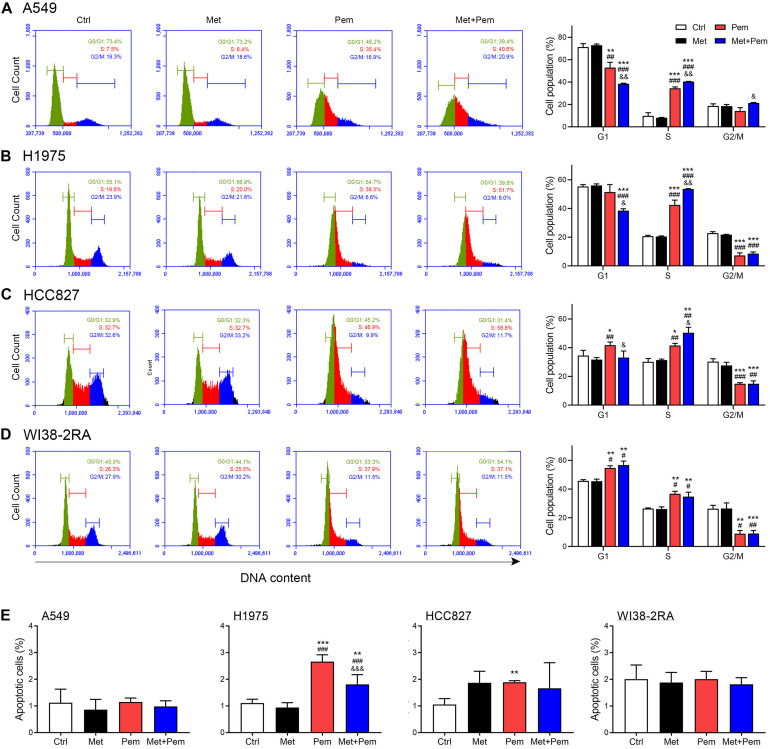
Additive cell cycle arrest and apoptosis effects of metformin and pemetrexed on the NSCLC and normal lung cell lines. Flow cytometric analysis and the cell cycle distribution of **(A)** A549, **(B)** H1975, **(C)** HCC827, and **(D)** WI38-2RA cells treated with metformin (0.2 mM) and/or pemetrexed (1 μM) for 48 h. (**A–D**, right panel) Quantification of cell cycle distribution. **(E)** Cytotoxicity of A549, H1975, HCC827, and WI38-2RA treated with the indicated concentration of metformin and/or pemetrexed for 48 h as assessed by flow cytometric analysis with Annexin V/propidium iodide (PI) staining. Percentages of Annexin V-positive cells (apoptosis cells population) are shown. The data are presented as the mean ± SD of those from three independent experiments with three technical replicates in each. *P < 0.05, **P < 0.01, and ***P < 0.001 compared with the Ctrl group; ^#^*P* < 0.05, ^##^*P* < 0.01, and ^###^*P* < 0.001 compared with the Met group; ^&^*P* < 0.05, ^&&^*P* < 0.01, and ^&&&^*P* < 0.001 compared with the Pem group.

Mounting evidence have shown the half maximal inhibitory concentration (IC50) of metformin and pemetrexed in NSCLC cell lines, the IC50 values at 48 h of metformin was 1–10 mM and the pemetrexed was 1.5–3.5 μM (Zhang et al., 2017). To investigate the potential mechanism by which lower dosage of metformin and pemetrexed influence NSCLC cell growth, we analyzed the cell cycle and apoptosis. In three NSCLC cell lines and normal lung cells (WI38-2RA), the flow cytometric analysis of propidium iodide (PI)-labeled cells showed that the cells accumulated in the S phase after single pemetrexed treatment (Figures 2A–D); however, in the combination of metformin and pemetrexed-treated NSCLC cells showed more significant cells arrested in the S phase than single pemetrexed treatment groups (Figures 2A–C). In the WI38-2RA cell, no significant change between combination treatment or single pemetrexed treatment group (Figure 2D).

In Figure 2E, we found that apoptosis rates of three NSCLC cell lines and normal lung cells treated with metformin alone (0.2 mM), pemetrexed alone (1 μM) or a combination were less than 3%. Although a statistical increase in H1975 apoptotic cells treated with single pemetrexed treatment and combination treatment compared to control group, a trend was not evident. Taken together, low dosage of metformin and pemetrexed showed extreme low cytotoxic effect on normal lung cells and three NSCLC cell lines. The combination treatment of metformin and pemetrexed exerted an additive antiproliferative effect in three NSCLC cell lines we used with either wild type epidermal growth factor receptor (EGFR; A549) or mutant type EGFR (H1975 and HCC827). Therefore, NSCLC cell line with wild type EGFR (A549) was used for the following experiments.

**FIGURE 3:**
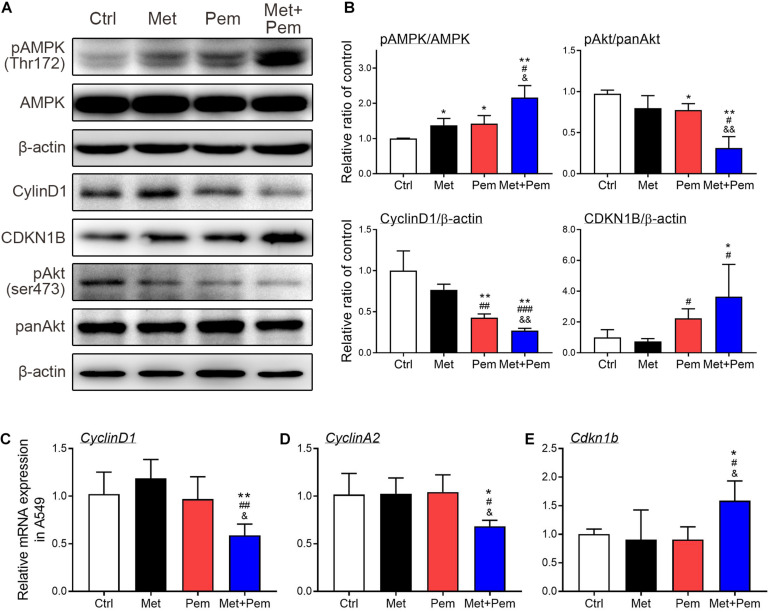
Additive cell cycle arrest of metformin and pemetrexed on A549 cell line through modulating AMPK-AKT signaling. **(A)** Western blotting was performed to detect the expression of pAMPK/AMPK, pAKT/panAKT, cyclin D1 and CDKN1B in the A549 cell line. Human β-actin was used as the normalization control. **(B)** Values were normalized to human β-actin levels and expressed relative to the control. RT-qPCR was performed to analyze **(C)** cyclin D1, **(D)** cyclin A2, and **(E)** CDKN1B. Values were normalized to the β-actin gene and expressed in relation to the control. Data are presented as the mean ± SD of those from three independent experiments with two technical replicates in each. *P < 0.05 and **P < 0.01 compared with the Ctrl group; ^#^*P* < 0.05, ^##^*P* < 0.01 and ^###^*P* < 0.001 compared with the Met group; ^&^*P* < 0.05 and ^&&^*P* < 0.01 compared with the Pem group.

Downregulation of cyclin D1, cyclin A2 and pAKT and upregulation of CDKN1B expression caused cell cycle arrest in the S phase (Cai et al., 2018). Western blot analysis showed that phosphorylated AMPK expression level significantly increased, which were further increased in the combination treatment groups. The expression level of phosphorylated AKT also significantly decreased in the combination treatment groups (Figures 3A,B). Hence, cell cycle regulatory molecules expression level also detected by using western blot and RT-qPCR analysis. Results showed that the combination of metformin and pemetrexed significantly downregulated cyclin D1 and cyclin A2 expression and upregulated CDKN1B expression, possibly through activated AMPK pathway to downregulate AKT signaling (Figure 3).

### Additive Antiangiogenic Effect of the Combination of Metformin and Pemetrexed in an *ex vivo* Chicken Embryo Chorioallantoic Membrane Assay

**FIGURE 4:**
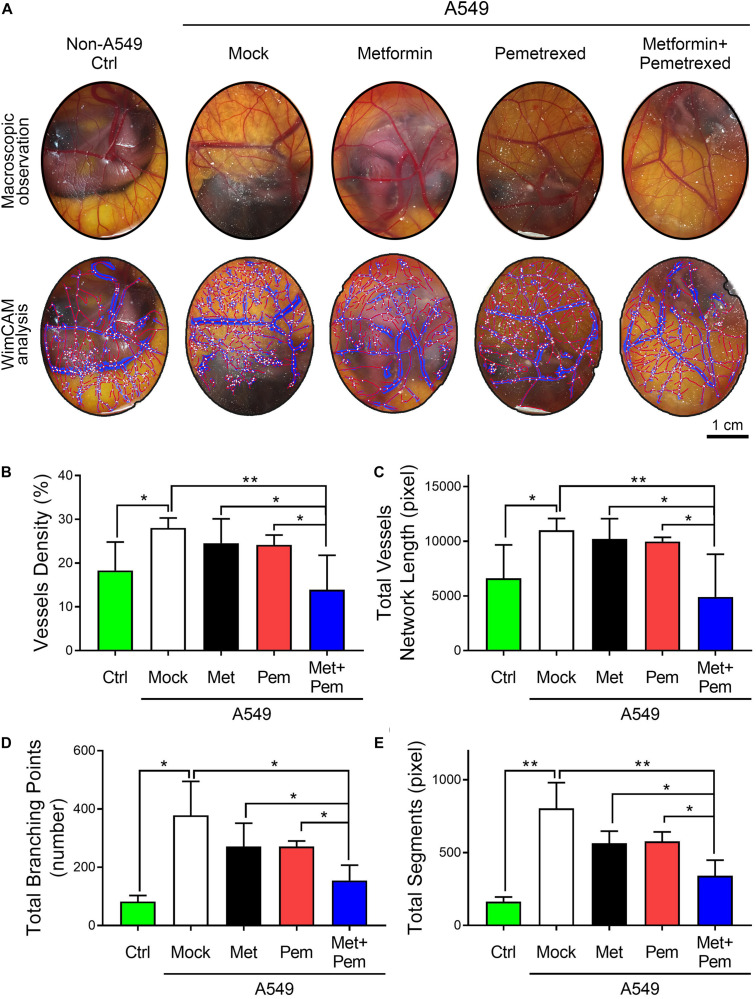
The combination of metformin with pemetrexed attenuated A549 cell-induced angiogenesis in a chick embryo chorioallantoic membrane (CAM) assay. **(A)** The upper panel shows the blood vessel plexus of 14-day-old chicken embryos following A549 xenograft metformin (0.1 mg/egg) and/or pemetrexed (0.02 mg/egg) treatment. The lower panel shows the WimCAM analysis results in which vessel structure (blue), vessel skeleton (red) and branching points (white) are marked. The statistical bar chart shows **(B)** blood vessel density, **(C)** total blood vessel network length, **(D)** number of branching points, and **(E)** total blood vessel segments for the control, mock and treatment groups. Scale bars = 1 cm. Data are presented as the mean ± SD. those from 3–4 independent experiments with two technical replicates in each. *P < 0.05 and **P < 0.01.

For angiogenesis models, the CAM assay is a popular choice. We chose chicken embryos and established A549 xenograft models in five groups, followed by the addition of metformin (0.1 mg/egg) and/or pemetrexed (0.02 mg/egg) separately. After 7 days of treatment, macroscopic observation showed that blood vessel density significantly decreased in the metformin and pemetrexed combination groups (Figure 4A, upper panel). WimCAM analysis (Figure 4A, lower panel) was used for quantitative analysis and provided a statistical bar chart for blood vessel density (Figure 4B), total blood vessel network length (Figure 4C), number of branching points (Figure 4D) and total blood vessel segments (Figure 4E) for the five groups (control, mock and three treatment groups). The results showed that the combination of metformin with pemetrexed significantly reduced angiogenesis and vessel growth compared with the mock or single treatment groups.

### *In vivo* Visualization and Quantification of the Additive Antitumor Growth Effect of the Combination of Metformin and Pemetrexed in an A549 Orthotopic Xenograft Model

**FIGURE 5:**
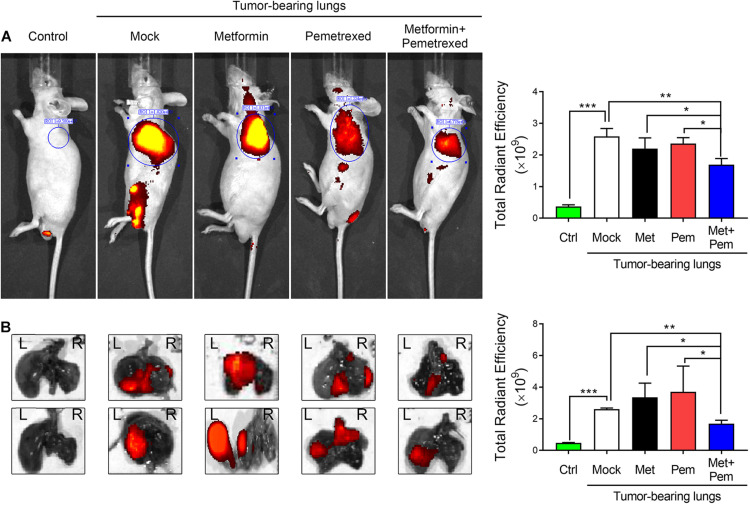
The combination of metformin with pemetrexed exerted additive inhibitory effects on A549 cell growth in a human orthotopic NSCLC xenograft model. An orthotopic lung tumor model was established *via* transpleural injection of 1 × 10^6^ fluorescently labeled A549 cells (iRFP-2A-Venus). After 4 weeks of A549 grafting, the mice were treated with metformin (Met; 250 mg/kg/day), pemetrexed (Pem; 150 mg/kg/twice a week) or both (Met + Pem). [**(A)** left panel] After a 3-week period of drug treatment, *in vivo* fluorescence images were acquired using an IVIS^®^ (Spectrum CT instrument) at the appropriate wavelength (iRFP: λ 650/720 nm). [**(A)** right panel] Statistical bar chart shows the total radiant efficiency of the iRFP signals, which was calculated using Living Image software. [**(B)** left panel] Whole lungs were excised from nude mice, and *ex vivo* fluorescence imaging was observed directly through the IVIS Spectrum system. [**(B)** right panel] Quantitative analysis of fluorescence intensity based on *ex vivo* images. All images were scaled to the same minimum and maxi-mum color values. The data are presented as the mean ± SD of those from 2–3 independent experiments with two technical replicates in each. *P < 0.05, **P < 0.01, and ***P < 0.001.

In our previous study, we established an A549 orthotopic xenograft model (Lai et al., 2016). After 4 weeks of A549 grafting, the mice were treated with metformin (Met; 250 mg/kg/day), pemetrexed (Pem; 150 mg/kg/twice a week), or both (Met + Pem) for 3 weeks. We recorded tumor size with the aid of IVIS (a Spectrum CT instrument) and quantitatively analyzed the total radiant efficiency of the region of interest (ROI) in each group (Figure 5A). This showed decreased total radiant efficiency in the combination treatment group (Figure 5A, left panel). After sacrifice of the mice, we performed *ex vivo* fluorescence imaging and observed the results directly through the IVIS Spectrum system (Figure 5B, left panel). The quantitative analysis showed a reduction in total radiant efficiency after the combination of metformin and pemetrexed treatment (Figure 5B, right panel).

### The Combination of Metformin With Pemetrexed Exerted Additive Inhibitory Effects on Pulmonary Tumorigenesis and Antiangiogenic Effects in an A549 Orthotopic Xenograft Model

**FIGURE 6:**
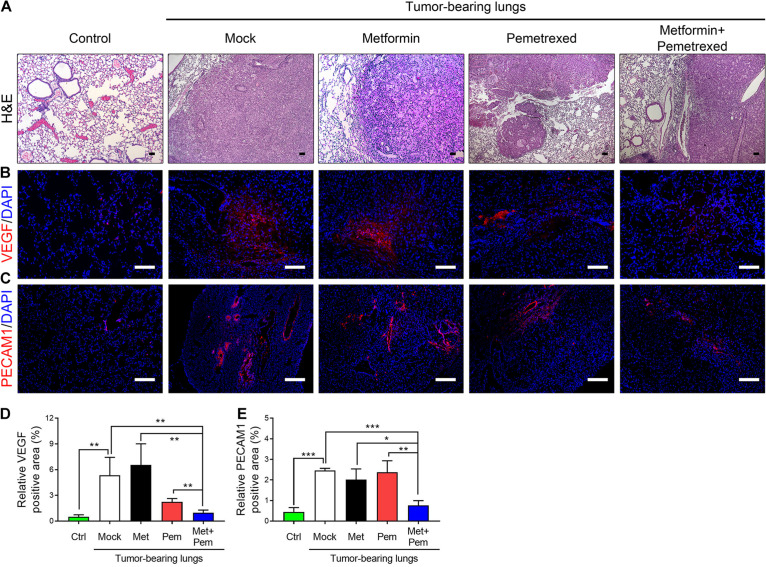
The combination of metformin with pemetrexed exerted additive inhibitory effects on pulmonary tumorigenesis in a human orthotopic NSCLC xenograft model. **(A)** H&E staining, immunofluorescence staining for **(B)** VEGF (red), **(C)** PECAM1 (red) and nuclei (DAPI; blue) in nude mouse lung tissues after a 3 week treatment period with metformin (Met; 250 mg/kg/day), pemetrexed (Pem; 150 mg/kg/twice a week) or both (Met + Pem) following a 4 week A549 cell xenograft. Scale bar = 100 μm. The area of **(D)** VEGF and **(E)** PECAM1 positive signal were calculated in three field from three independent experiments and represented as% of positive area. The data are presented as the mean ± SD of those from 2–3 independent experiments with two technical replicates in each. *P < 0.05, **P < 0.01, and ***P < 0.001.

We further analyzed the angiogenesis associated indicators involved in tumorigenesis by our A549 orthotopic NSCLC xenograft model. After 3 weeks of treatment, we checked the histopathologic findings, including hematoxylin and eosin (H&E) staining (Figure 6A) and immunofluorescence (IF) staining of VEGF and PECAM-1, a marker of endothelial cells (Figures 6B–E). The percentage of VEGF-positive area significantly increased in the A549 tumor-bearing lung (mock group) and single metformin treatment group; however, single pemetrexed treatment decreased the percentage of VEGF-positive area, which were further decreased in the combination treatment groups (Figure 6D). In addition, the percentage of PECAM-1-positive area significantly higher in the mock and single treatment groups; however, a significant less PECAM-1-positive area was detected in the combination treatment group, indicating less presence of endothelium of blood vessels formation within the lung tumor site (Figure 6E).

**FIGURE 7:**
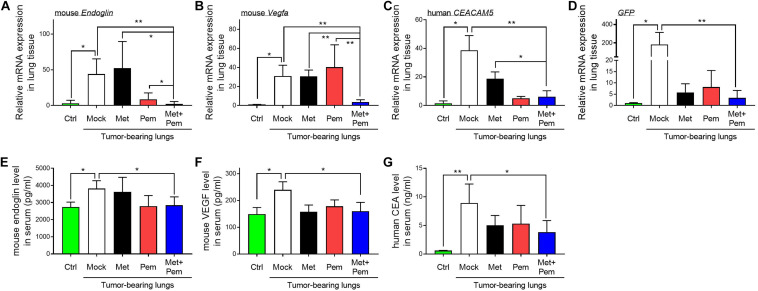
Molecule expression related to the metformin/pemetrexed additive inhibitory effects on pulmonary tumorigenesis and angiogenesis in a human orthotopic NSCLC xenograft model. RT-qPCR was performed to analyze the expression of angiogenic indicators **(A)** *Endoglin* and **(B)** *Vegfa*, tumor marker **(C)** carcinoembryonic antigen (*CEACAM5*), and **(D)** GFP in the lung tissues of mice that received different treatments. Values were normalized to GAPDH levels and are expressed relative to the control group. The expression levels of **(E)** Endoglin, **(F)** VEGF and **(G)** human CEA in the serum of the mice were determined using ELISA kits. The data are presented as the mean ± SD of those from 2–3 independent experiments with two technical replicates in each. *P < 0.05 and **P < 0.01.

Moreover, all the mRNA expression levels of the angiogenic indicators *Endoglin* (Figure 7A) and *Vegfa* (Figure 7B), along with those of tumor marker carcinoembryonic antigen (CEACAM5) (Figure 7C) and green fluorescent protein (GFP) (Figure 7D), in the lung tissues of mice showed significant downregulation in the metformin and pemetrexed combination treatment group. Among the different indicators, we proved the obvious additive effect of combined metformin and pemetrexed on VEGFA expression. This indicates that the combination of metformin and pemetrexed truly targeted the angiogenesis pathway. In addition, we checked the expression of Endoglin (Figure 7E), VEGF (Figure 7F) and human CEA (Figure 7G) in the serum of the mice by ELISA kits. Taken together, the combination of metformin and pemetrexed had an impact on reducing the expression of the above indicators compared with the mock and single treatment groups.

## Discussion

From the experimental results, we found that the combination of metformin and pemetrexed exerted additive antitumor effects *via in vitro*, *ex vivo*, and *in vivo* models. In three NSCLC cell lines model, when metformin was added to pemetrexed, an antiproliferative effect was observed through the inhibition of colony formation (Figure 1), as well as an induced cell cycle arrest (G1/S phase) (Figure 2) through regulating the AMPK/AKT signaling pathway (Figure 3). In a chicken embryo chorioallantoic membrane (CAM) assay (*ex vivo* model), metformin combined with pemetrexed was shown to be antiangiogenic by reducing vessel density, network formation and microenvironment formation. The antiangiogenic effect was also validated in our orthotopic NSCLC xenograft model, which included real-time imaging (IVIS) and *in vivo* analysis of VEGF-associated angiogenic indicators.

Previous literature has reviewed the scope of the potential antitumor effect of the use of metformin alone or as an adjuvant to other anticancer modalities, including chemotherapeutic reagents (Peng et al., 2017; Levy and Doyen, 2018; Fatehi Hassanabad and MacQueen, 2020). Activation of the AMPK-dependent pathway has been extensively discussed (Zakikhani et al., 2006; Emami Riedmaier et al., 2013; Sinnett-Smith et al., 2013). Once AMPK is activated, it phosphorylates and activates tuberous sclerosis complex 2 (TSC2). This leads to the subsequent negative regulation of the mTOR pathway with the help of liver kinase B1 (LKB1) to induce an antiproliferative effect (Shaw et al., 2005). The mTOR pathway is the core for cell proliferation and protein formation (Sinnett-Smith et al., 2013). It can also be upregulated *via* the phosphoinositide 3-kinase/protein kinase B/AKT (PI3K/PKB/AKT) pathway. Metformin can target the mTOR pathway by activating AMPK to inhibit tumorigenesis by decreasing cancer cell proliferation (Gwinn et al., 2008; Emami Riedmaier et al., 2013; Sinnett-Smith et al., 2013). In cell cycle regulation, Kato et al. (2012) and Rattan et al. (2011) showed that metformin can reduce the expression of cyclin D1, CDK4 and CDK6 in ovarian and gastric cancer cell lines and xenograft models. The additional inhibition of the expression of cyclin D1 and retinoblastoma protein was also mentioned (Ben Sahra et al., 2008).

Metformin can also enhance p53-mediated cell cycle arrest *via* the AMPK-related apoptosis pathway (Buzzai et al., 2007; Emami Riedmaier et al., 2013). In an *in vitro* study of A549, H1975, and HCC827 lung cancer cell lines, Zhang et al. (2017) found that metformin combined with pemetrexed not only inhibited cell proliferation but also induced cell apoptosis, as validated by western blot analysis of reduced levels of an antiapoptotic molecule (Bcl-2) and elevated levels of a proapoptotic molecule (Bax). In our study, we showed a similar result as Gong et al. described (Steiner et al., 2007) that the single uses of low dosage of metformin show no antiproliferative or apoptotic effect on A549 cells. However, the combination of low dosage of metformin and pemetrexed reached an additive antiproliferative effect by reducing the expression of cyclin D1 and A2 and augmenting CDKN1B expression. The additive effect was based on regulation of the cell cycle involved in the G1/S phase.

Angiogenesis is crucial for tumor growth, development and distal metastasis (Tosetti et al., 2002; Weis and Cheresh, 2011; Albini et al., 2012; Wang et al., 2020). As the tumor enlarges, proangiogenic factors need to be established when exposed to a hypoxic environment. The key element for this is vascular endothelial growth factor. The VEGF family is composed of four subgroups: VEGF-A, VEGF-B, VEGF-C, and VEGF-D. VEGF-A has potent efficacy and has four isoforms, and VEGF-A165 is the dominant isoform. The angiogenesis process and tumor development are related to VEGF-A165 formation. In our study, the combination of metformin with pemetrexed reduced VEGF-A expression. This also showed inhibition of angiogenesis by immunohistochemistry staining for VEGF and PECAM1 (Figures 6B–E), similar to the finding of Rattan et al. (2011). Joe et al. (2015) found that metformin possesses an antiangiogenic effect in mouse oxygen-induced retinopathy and is undermodulated by reducing the vascular endothelial growth factor receptor Flk-1. Other research found that metformin can alter and regulate the downregulation of microRNA (miR)-21 by upregulating TGF-β to enhance the antiangiogenic mechanism (Wang et al., 2020). Further exploration of the up-stream regulatory mechanism is indeed needed.

Endoglin, a cell membrane glycoprotein that represents the endothelium in angiogenesis, can serve as tumor metastasis and prognostic factors (Fonsatti et al., 2003a, b; Wang et al., 2018). In our study (Figures 7A–E), combined metformin and pemetrexed revealed the trend of reduced Endoglin expression in an orthotopic xenograft model *via* RT-qPCR and ELISA analysis. We performed a CAM assay (*ex vivo* model) to clearly observe the macroscopic angiogenic structure and evaluated vessel formation by WimCAM analysis. We reconfirmed the serial validation of the antiangiogenic effects of the combination of metformin and pemetrexed from *ex vivo* to *in vivo* orthotopic xenograft models. In our designed A549 orthotopic xenograft model, we discovered and recorded tumor formation by the IVIS method. Transpleural injection into primary lung tumorigenesis instead of a subcutaneous tumor model helped us to more closely monitor and audit the therapeutic response *in situ*, along with the great advantage of real-time image capture (Chen et al., 2017).

When metformin is used as adjuvant therapy for chemotherapeutic agents in lung cancer, it can have beneficial antitumor effects (Iliopoulos et al., 2011; Lin et al., 2013; Tseng et al., 2013; Parikh et al., 2019). It has shown potential efficacy and clinical tolerability. Parikh et al. (2017) proposed a pilot study of a phase II trial for the evaluation of metformin therapy combined with a first-line chemotherapeutic regimen (carboplatin and pemetrexed) in advanced non-diabetic NSCLC. A lower toxicity, low cost and good tolerance of metformin were observed during the dosage titration. Despite the small sample size, the study revealed the feasibility of applying metformin combined with pemetrexed in lung cancer patients (Parikh et al., 2017). Another randomized phase II study of chemotherapy (paclitaxel/carboplatin/bevacizumab) with/without metformin in chemotherapy-naive advanced or metastatic NSCLC groups showed an improved progression-free survival (PFS) (9.6 vs. 6.7 months, *P* = 0.024), though no overall survival (OS) significant change was observed (Marrone et al., 2018). In a recent FAME trial, metformin and a fasting-mimicking diet (FMD) were exploited for their potential efficacy when combined with platinum-pemetrexed chemotherapy in advanced LKB1-inactivated lung adenocarcinoma. This trial is still under way. The expected goal is to improve the median PFS from 7.6 months (chemotherapy alone) to 12 months (combined with metformin) (Vernieri et al., 2019).

Finally, our research highlights the related antitumor mechanism provided by metformin and pemetrexed in combination. In addition to AMPK-dependent pathway-related antiproliferative effects, we also showed antiangiogenic clues for potential additional and helpful support for advanced research involved in combination therapy.

## Conclusion

In this study, we proved the additive antiproliferative and antiangiogenic effects of the combination of metformin and pemetrexed in a lung cancer orthotopic xenograft model. From *in vitro*, *ex vivo*, and *in vivo* models, we found a new approach for investigating the antitumor mechanisms of metformin and pemetrexed. Further large-scale randomized trial aimed at lung cancer therapy, including a combination of metformin and pemetrexed, are encouraged and anticipated.

## Data Availability Statement

The original contributions presented in the study are included in the article/Supplementary Material, further inquiries can be directed to the corresponding author/s.

## Ethics Statement

The animal experiments were approved by the Institutional Animal Care and Use Committee of National Chung Hsing University (IACUC no. 105–070).

## Author Contributions

J-LW, Y-WL, Y-TT, C-CY, and C-MC: conceptualization. J-LW, Y-WL, and Y-TT: methodology and visualization. J-LW: software. J-LW, TS, and Y-WL: validation. Y-WL, TS, and C-MC: formal analysis. J-LW, Y-WL, and Y-CC: investigation. Y-TT and Y-AT: resources. Y-AT, Y-WL, and C-MC: data curation. J-LW and Y-WL: writing original draft preparation. C-CY and C-MC: writing review and editing. C-MC: supervision. Y-CC and Y-WL: project administration. J-LW and C-MC: funding acquisition. All authors have read and agreed to the published version of the manuscript.

## Conflict of Interest

The authors declare that the research was conducted in the absence of any commercial or financial relationships that could be construed as a potential conflict of interest.

## Supplementary Material

Supplementary Figure 1 Whole western blot images with densitometry readings. **(A–H)** Whole western blot images with all molecular weight markers depicted in Figure 3A. Molecular weight markers are indicated. The intensity values and calculated ratios of each targeted band of the Western blots depicted in **(A–H)**.

Supplementary Figure 2 All IVIS images of whole animals and isolated lungs used within the main text of Figure 5.
