# Supplementary figures and images for: Additive Antiproliferative and Antiangiogenic Effects of Metformin and Pemetrexed in a Non-Small-Cell Lung Cancer Xenograft Model
Source: Front Cell Dev Biol. 2021 Jun 21;9:688062. doi: 10.3389/fcell.2021.688062 (PMC8255984; doi:10.3389/fcell.2021.688062)

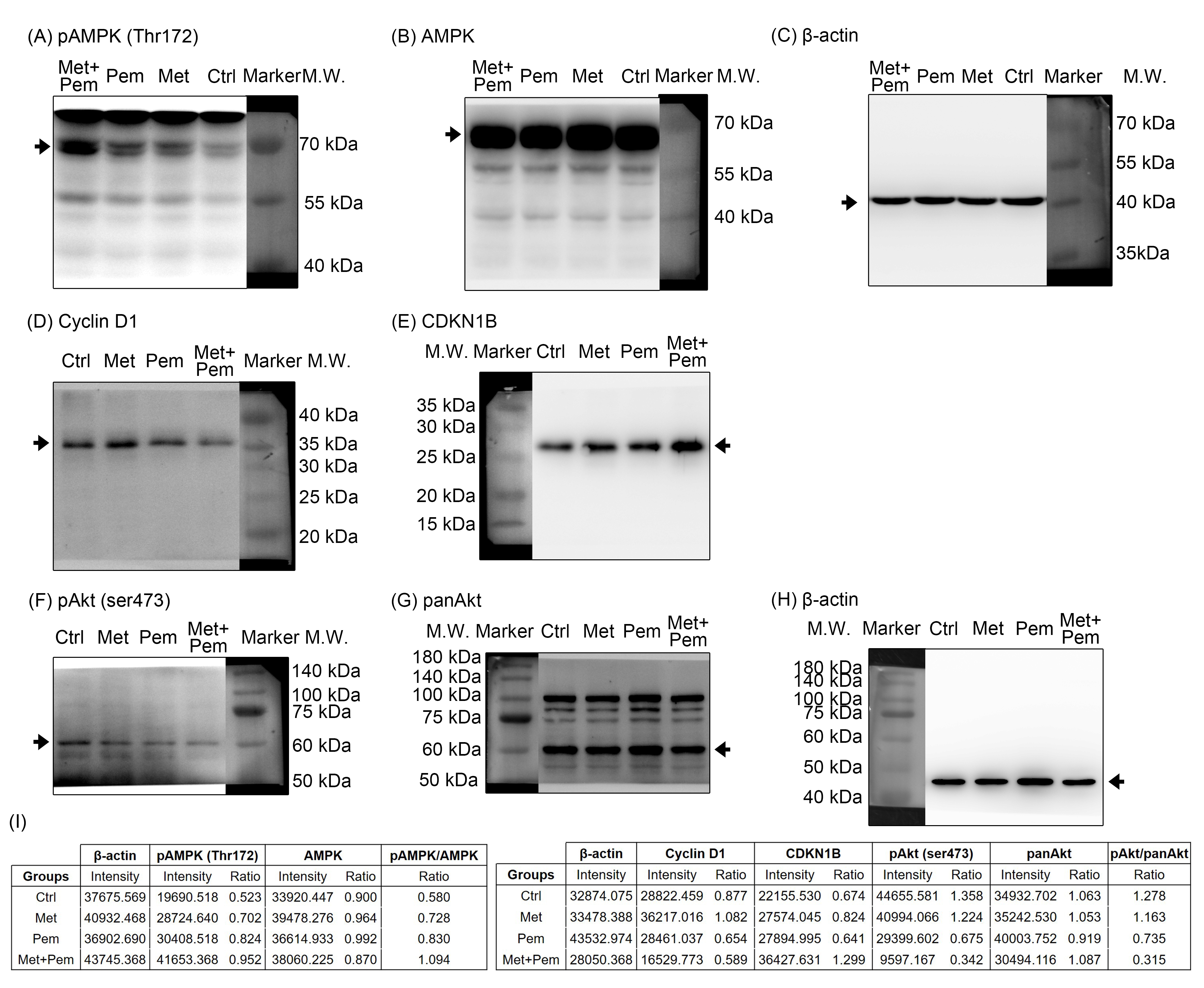

Supplement: Supplementary Figure 1 — Whole western blot images with densitometry readings. (A–H) Whole western blot images with all molecular weight markers depicted in Figure 3A. Molecular weight markers are indicated. The intensity values and calculated ratios of each targeted band of the Western blots depicted in (A–H). [file Image_1.JPEG]

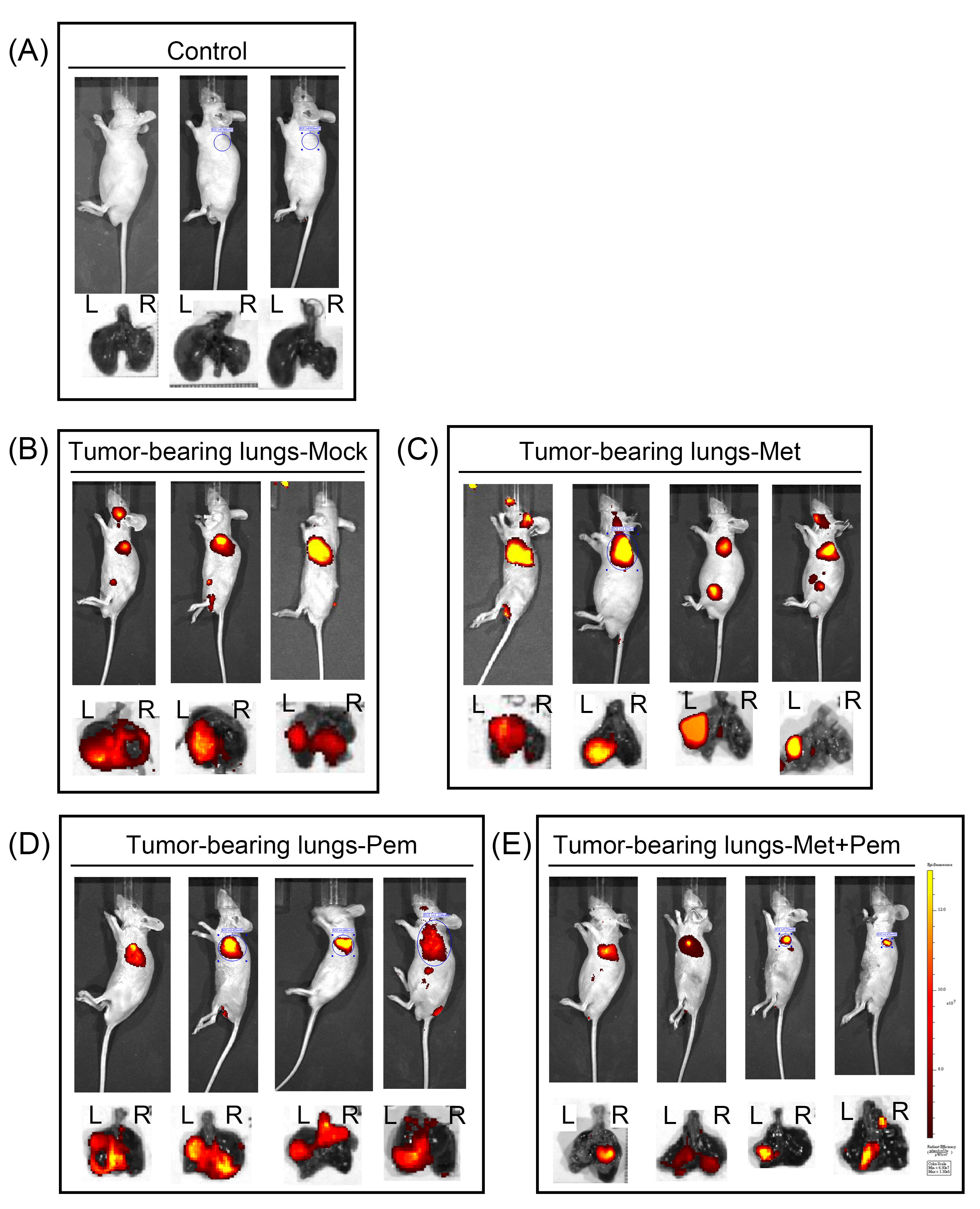

Supplement: Supplementary Figure 2 — All IVIS images of whole animals and isolated lungs used within the main text of Figure 5. [file Image_2.JPEG]
